# Antiviral activity of the EB peptide against zoonotic poxviruses

**DOI:** 10.1186/1743-422X-9-6

**Published:** 2012-01-06

**Authors:** Sharon E Altmann, Curtis R Brandt, Peter B Jahrling, Joseph E Blaney

**Affiliations:** 1 Emerging Viral Pathogens Section, National Institute of Allergy and Infectious Diseases, National Institutes of Health, Bethesda, MD 20892, USA; 2 Departments of Ophthalmology & Visual Sciences and Medical Microbiology & Immunology, University of Wisconsin, Madison, WI 53706, USA; 3 Integrated Research Facility, National Institute of Allergy and Infectious Diseases, National Institutes of Health, Fort Detrick, MD 21702, USA

**Keywords:** EB peptide, vaccinia, cowpox, monkeypox, poxvirus entry, poxvirus attachment

## Abstract

**Background:**

The EB peptide is a 20-mer that was previously shown to have broad spectrum *in vitro *activity against several unrelated viruses, including highly pathogenic avian influenza, herpes simplex virus type I, and vaccinia, the prototypic orthopoxvirus. To expand on this work, we evaluated EB for *in vitro *activity against the zoonotic orthopoxviruses cowpox and monkeypox and for *in vivo *activity in mice against vaccinia and cowpox.

**Findings:**

In yield reduction assays, EB had an EC_50 _of 26.7 μM against cowpox and 4.4 μM against monkeypox. The EC_50 _for plaque reduction was 26.3 μM against cowpox and 48.6 μM against monkeypox. A scrambled peptide had no inhibitory activity against either virus. EB inhibited cowpox *in vitro *by disrupting virus entry, as evidenced by a reduction of the release of virus cores into the cytoplasm. Monkeypox was also inhibited *in vitro *by EB, but at the attachment stage of infection. EB showed protective activity in mice infected intranasally with vaccinia when co-administered with the virus, but had no effect when administered prophylactically one day prior to infection or therapeutically one day post-infection. EB had no *in vivo *activity against cowpox in mice.

**Conclusions:**

While EB did demonstrate some *in vivo *efficacy against vaccinia in mice, the limited conditions under which it was effective against vaccinia and lack of activity against cowpox suggest EB may be more useful for studying orthopoxvirus entry and attachment *in vitro *than as a therapeutic against orthopoxviruses *in vivo*.

## Findings

The EB peptide (NH_2_- RRKKAAVALLPAVLLALLAP-COOH) is a 20-mer derived from the signal peptide of the human FGF4 protein [1] and was originally identified as an inhibitor of herpes simplex virus entry [2]. Subsequent work demonstrated that EB was active against several strains of influenza virus both *in vitro *and *in vivo *[3], with a minimum of 13 core amino acids being identified as necessary to block influenza attachment to host cells [4]. EB was also identified as an inhibitor of Vaccinia virus entry into host cells *in vitro *[5]. This broad range of antiviral activity against a number of unrelated viruses, in combination with low *in vivo *toxicity [6], makes EB an attractive candidate for a broad-spectrum antiviral therapy.

Vaccinia virus (VACV) is the most-studied member of the orthopoxviruses, a genus of large, double-stranded DNA virus whose most notorious member, Variola virus, the etiologic agent of smallpox, was declared eradicated in 1980 [7]. Vaccinia virus infection typically results in a self-limiting infection in immunocompetent individuals; the closely-related cowpox (CPXV) and monkeypox (MPXV) viruses, however, are both considered to be emerging zoonotic agents [8,9] with the potential to cause serious morbidity and, in the case of MPXV, mortality in infected hosts [10]. There are currently no FDA-approved therapeutics for treating orthopoxvirus infections, and vaccination is counter-indicated for an increasingly large percentage of the global population, highlighting the need for novel therapeutic options. The relatively low global incidence of severe orthopoxvirus disease, however, makes identifying broad spectrum drugs with activity against a number of unrelated viruses, including the orthopoxviruses, economically advantageous. To expand upon the initial characterization of EB peptide anti-orthopoxvirus activity, the goals of this work were to test EB for efficacy against CPXV and MPXV *in vitro*, to begin to determine the mechanism for any inhibition observed, and to test EB for *in vivo *activity in two well-characterized mouse models of orthopoxvirus disease, VACV and CPXV.

**Figure 1:**
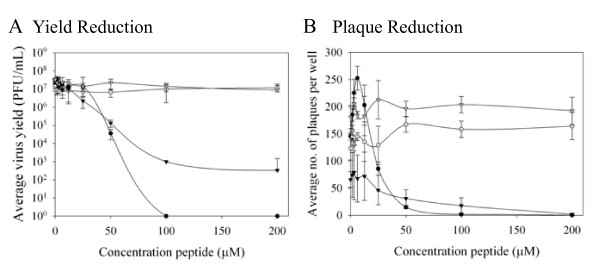
**EB inhibits CPXV and MPXV *in vitro***. A) Yield reduction assay. BSC-1 cells in 24-well plates were infected at a m.o.i. of 0.01 with the indicated virus or with virus pre-treated for one hour with EB. Three days post-infection, cells were harvested by scraping, lysed by repeated cycles of freezing and thawing, and titered on BSC-1 cells. Data represent the average of three independent assays. B) Plaque reduction assay. BSC-1 cells in 6-well plates were infected with approximately 100 pfu virus/well with or without peptide. One hr post-infection, the wells were overlaid with 2% agarose. Plates were fixed 3 days post-infection and plaques were enumerated. Data represent the average of four independent assays. Circles, CPXV; triangles, MPXV. Closed symbols, EB; opens symbols, EBX.

To determine whether EB had antiviral activity against CPXV (Brighton strain) and MPXV (Zaire 76 strain), the effect of increasing concentrations of the peptide (American Peptide Company, Inc., Vista, CA) on virus yield was determined (Figure 1A). All peptides used were synthesized with all dextral amino acids to reduce proteolysis. The 50% effective concentration (EC_50_) of EB against CPXV was 26.7 μM, while MPXV was more sensitive to EB with an EC_50 _of 4.4 μM. The EBX peptide (NH_2_-RRKLLAALPLVLAAPLAVLA-COOH), a derivative of EB with a scrambled signal sequence failed to significantly reduce CPXV or MPXV yield, indicating that the inhibition seen with the parent peptide was sequence-specific. EB was also active against CPXV and MPXV in plaque reduction assays, with EC_50 _values of 26.3 and 48.6 μM, respectively, whereas EBX had no effect on either virus (Figure 1B). The different susceptibilities of CPXV and MPXV to EB in these two assays suggested that EB was acting differently on the two viruses. As EBX showed no activity against either virus, it was not included in further assays.

**Figure 2:**
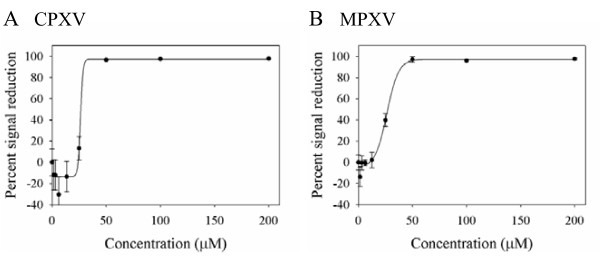
**EB reduces gene expression in CPXV and MPXV**. Triplicate wells of BSC-1 cells were infected with recombinant CPXV (A) or recombinant MPXV (B) expressing GFP under the control of an artificial early/late promoter at a m.o.i. of 1 in the presence of EB for 1 hr at 4°C, rinsed 3 times, and treated with peptide-containing media. Twenty-four hours post-infection, GFP intensity was measured at 520 nm. Data are representative of three independent assays.

EB was next tested for its ability to reduce gene expression during CPXV and MPXV infections (Figure 2). BSC-1 cells were infected with recombinant CPXV or MPXV expressing GFP under the control of a synthetic early/late promoter in the presence of increasing concentrations of EB. The EB peptide reduced GFP expression by both viruses, with an EC_50 _of 26.7 μM against CPXV and 27.6 μM against MPXV, indicating that EB acted upstream of gene expression to inhibit both viruses.

**Figure 3:**
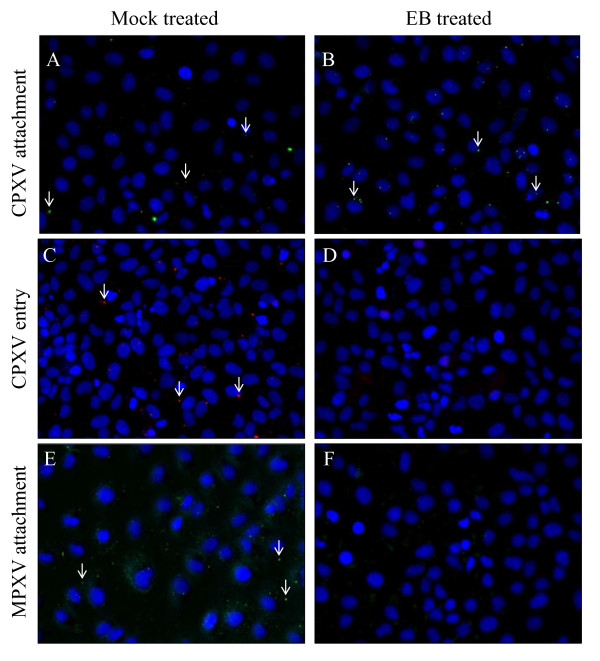
**Effect of EB on CPXV and MPXV attachment and entry**. CPXV (A, B, C, D) or MPXV (E, F) in the presence (B, D, F) or absence (A, C, E) of 100 μM EB was allowed to attach to BSC-1 cells for 1 hr at room temperature. Samples for attachment staining (A, B, E, F) were fixed with 4% paraformaldehyde, quenched for 5 minutes with 100 mM glycine, blocked with 10% FBS in PBS, and stained with 1:200 anti-VACV antibody (V0500-11D, US Biologial). Samples for entry staining (C, D) were incubated 1 hr at 37°C prior to PFA fixation, glycine quenching, permeabilization with 0.1% saponin, and staining with anti-core antibody (R236). Nuclei were counterstained with DAPI (blue). Arrows indicate selected virions (green) or virus cores (red).

Orthopoxvirus cores are only accessible to antibodies after being released into the cytoplasm, allowing for the differentiation between attached and entered virus using virion-specific or core-specific antibodies, respectively [11]. To examine whether EB was disrupting virus attachment or entry into the cells, immunofluorescent microscopy was used to quantify the number of attached and entered viruses per cell for 100 cells in the absence or presence of 100 μM EB. The presence of EB had no significant effect on the number of CPXV virions attached to cells (5.4 virions/cell, untreated *vs*.4.7 virions/cell, treated, *p *> 0.01; Figure 3A*vs*. 3B) but significantly reduced virus entry (1.3 cores/cell, untreated *vs*. 0.3 cores/cell, treated, *p *< 0.0001; Figure 3C*vs*. 3D). These data are similar to what has been reported with VACV, where virus attachment was unaffected by peptide treatment but core release was significantly reduced [5]. In contrast, MXPV attachment was significantly inhibited by EB (3.2 virions/cell, untreated *vs*. 0.9 virions/cell, treated, *p *< 0.001; Figure 3E*vs*. 3F). Based on the observed disruption of virus attachment, MPXV entry was not examined. This different target of inhibition is consistent with the different pattern of EB susceptibility displayed by MPXV in the yield reduction assay compared to that demonstrated by CPXV and VACV. As inhibition of both CPXV and MPXV by EB occurs extracellularly, it is unlikely that the peptide's previously-described ability to inhibit NF-κB signaling [1] is involved in its anti-orthopoxvirus activity.

**Figure 4:**
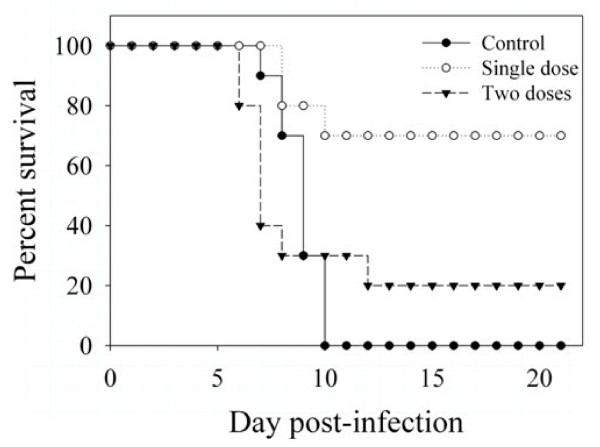
***In vivo *activity of EB**. Ten female BALB/c mice were inoculated intranasally with 20 μL containing 10^5 ^PFU VACV (Control) or 10^5 ^PFU VACV mixed with10 mg/kg EB (Single dose) in PBS on day 0. One additional group of animals received a second dose of EB intranasally on day 3 (Two doses). Animal survival was monitored for 21 days, and mice were euthanized when they met established endpoint criteria.

EB has shown efficacy in an *in vivo *model of lethal influenza infection when added at the time of infection or daily starting 6 hours post-infection [3] but has not been tested against any orthopoxviruses. As there is currently no well-characterized mouse model for MPXV infection available, EB was only tested against VACV (strain WR) and CPXV (strain Brighton) *in vivo*. To test EB for *in vivo *efficacy against VACV, EB (10 mg/kg) was administered intranasally concurrently with 10^5 ^PFU of VACV (strain WR). Seventy percent of infected animals treated with a single dose of EB at the time of infection survived to study end, compared to no survivors in untreated animals (Figure 4). Two doses of EB (day 0, day 3) were less effective than a single dose, with only 20% of animals surviving. Animals receiving 2 doses met clinical endpoint criteria slightly earlier than control animals, which may suggest that further intranasal treatment exacerbated disease. EB showed no efficacy when administered prophylactically (1 day pre-infection) or therapeutically (1 day post-infection) to VACV-infected mice (data not shown). EB also demonstrated no efficacy when administered prophylactically, at the time of infection, or therapeutically to mice infected intranasally with CPXV (data not shown).

In summary, we were able to demonstrate *in vivo *efficacy of EB against VACV but not CPXV, despite both viruses having similar sensitivities to the peptide *in vitro*. The reasons for this difference are as yet unclear. EB was only effective against VACV when co-administered with the virus, suggesting that the peptide needed direct interaction with the virus to be effective. EB self-associates in micelle-like structures at high concentrations and in high ionic buffers [12], a property which could influence bioavailability. It is possible that the *in vivo *anti-orthopoxvirus activity could therefore be improved by changing the vehicle used for delivery.

Most intriguing is the observation that EB inhibited attachment by MPXV but blocked entry by CPXV, as these processes are generally believed to be highly conserved between the orthopoxviruses. While EB has now been shown to have activity *in vivo *against VACV and influenza viruses [3], its greatest potential with orthopoxviruses may be as a novel *in vitro *tool to study the poorly-characterized early steps in infection. To date, over a dozen viral proteins are believed to be involved in orthopoxvirus attachment and entry [13-25]. The precise mechanisms of attachment and entry, however, have yet to be elucidated. Identification of the precise targets of the interaction between EB and VACV, CPXV, and MPXV could help identify key amino acids or structural features necessary for these processes and identify targets for novel inhibitors of infection.

## Competing interests

The authors declare that they have no competing interests.

## Authors' contributions

SEA carried out the *in vitro *and *in vivo *experiments, and SEA and JEB drafted the manuscript. SEA, CRB, PBJ and JEB participated in study design. All authors read and approved the final manuscript.
